# Small, Dense LDL Particles Predict Changes in Intima Media Thickness and Insulin Resistance in Men with Type 2 Diabetes and Prediabetes – A Prospective Cohort Study

**DOI:** 10.1371/journal.pone.0072763

**Published:** 2013-08-08

**Authors:** Philipp A. Gerber, Christoph Thalhammer, Christian Schmied, Silviana Spring, Beatrice Amann-Vesti, Giatgen A. Spinas, Kaspar Berneis

**Affiliations:** 1 Division of Endocrinology, Diabetes and Clinical Nutrition, University Hospital, Zürich, Zurich, Switzerland; 2 Division of Angiology, University Hospital, Zürich, Zurich, Switzerland; 3 Division of Cardiology, University Hospital, Zürich, Zurich, Switzerland; 4 Competence Centre for Systems Physiology and Metabolic Diseases, Zürich, Switzerland; 5 Centre for Integrative Human Physiology, Zürich, Switzerland; University of Tor Vergata, Italy

## Abstract

The association of small, dense low-density lipoprotein (sdLDL) particles with an increased cardiovascular risk is well established. However, its predictive value with regard to glucose metabolism and arterial disease in patients with type 2 diabetes has not been thoroughly investigated. We conducted a prospective longitudinal cohort study in patients with (pre)diabetes who were seen at baseline and after two years. sdLDL particles were determined by gradient gel electrophoresis. Insulin resistance was estimated by using the homeostatic model assessment 2 (HOMA2). Intima media thickness (IMT) and flow-mediated dilation (FMD) were assessed by ultrasound measurements. Fifty-nine patients (mean age 63.0 ± 12.2 years) were enrolled and 39 were seen at follow-up. IMT increased in the whole cohort during follow-up. The change in IMT was predicted by the proportion of sdLDL particles at baseline (p=0.03), and the change in FMD was predicted by LDL-cholesterol levels at baseline (p=0.049). HOMA2 and changes in HOMA2 correlated with the proportion of sdLDL particles and changes in this proportion, respectively (p<0.05 for both). Serum resistin levels increased in parallel with the increasing sdLDL particle number, while serum adiponectin increased only in patients with unaltered sdLDL particle number at follow-up (p<0.01 for both). In conclusion, the proportion of small, dense LDL particles and changes in this proportion are predictive of changes in intima media thickness and insulin resistance, and are closely associated with other determinants of an adverse metabolic status. Thus, this parameter extends the individual risk assessment beyond the limitations of traditional risk markers in patients with dysglycemia.

## Introduction

The risk of cardiovascular events in individuals with impaired glucose metabolism is similar to patients with a history of a prior cardiovascular event [1] and dysglycemia is associated with parameters of vascular damage [2]. However, increasing hyperglycemia in type 2 diabetes does not contribute to the cardiovascular risk to the same extent as it does in type 1 diabetes [3], pointing to the importance of nonglycemic related risk factors belonging to the “metabolic syndrome”. Numerous studies could demonstrate an increased cardiovascular risk in patients with metabolic syndrome prior to the development of overt hyperglycemia [4,5]. Likewise, patients with type 2 diabetes or metabolic syndrome have an increased cardiovascular risk despite optimal control of other risk factors as low-density lipoprotein cholesterol (LDL-C) [6].

In the context of the shortcomings of commonly assessed risk factors in individuals with features of the metabolic syndrome, the characterization and subclassification of LDL particles emerged as a tool that may offer a better risk prediction. An increase in sdLDL (small, dense LDL particles, class III and IV) is closely associated with an increased cardiovascular risk, independently of the traditional risk factors both in patients with [7–9] and without [10–12] diabetes or metabolic syndrome. Individual LDL particle profiles normally cluster into two patterns of LDL size distribution: The majority of profiles demonstrates a predominance of large or medium sized LDL particles (LDL pattern A), whereas a substantial minority exhibits the LDL pattern B with a higher proportion of smaller LDL particles [13].

Several aspects contribute to the significance of sdLDL in patients with the metabolic syndrome. The formation of sdLDL particles seems to be favoured in the presence of insulin resistance and hypertriglyceridemia [14,15]. Furthermore, there is growing evidence that sdLDL not only are more susceptible to oxidative modification [16], but also more prone to glycation [17] which further aggravates their atherogenicity in a hyperglycemic environment.

In several studies the association of sdLDL with actual insulin resistance and cardiovascular risk factors has been tested in a cross-sectional manner in patients with disturbed glucose metabolism; however, there is not much data about the longitudinal predictive value of these particles. Therefore, this study was designed to assess the relationship between sdLDL and parameters associated with insulin resistance and the metabolic syndrome including markers for atherosclerosis during a long-term follow-up in this population.

## Materials and Methods

### Study protocol

A cohort of 59 patients consulting the outpatient clinic of the Division of Endocrinology, Diabetes and Clinical Nutrition or the Division of Cardiology of the University Hospital, Zurich were included in a prospective study. In order to avoid gender related differences in outcome, the study was restricted to male patients. After written informed consent was given, subjects underwent physical examination and blood tests, and were asked to fill in a questionnaire on personal and medical data, including age, past medical history and current medication. The adopted procedures were in agreement with the Helsinki Declaration of 1975, as revised in 1983. The study was approved by the Ethics Committee of the Canton of Zürich, Switzerland and registered at clinicaltrials.gov (NCT01584856).

Inclusion criteria were male gender, impaired fasting glucose or type 2 diabetes according to the ADA criteria [18] and BMI >25 kg/m^2^ as well as given informed consent. Exclusion criteria were HbA1c >9.0%, insulin therapy, fasting glucose >11mmol/l, total cholesterol >6.5 mmol/l or fasting triglycerides >2.5 mmol/l, malignant or severe renal, hepatic, pulmonary, neurological or psychiatric disease, alcohol or drug abuse and HIV infection.

After inclusion, patients were seen for a first visit within a few weeks and after two years they were invited for the follow-up visit.

### Medical history and anthropometric measurements

Body weight was measured to the nearest 100 gram, height to the nearest centimetre. BMI (body mass index) was calculated as weight/height^2^ (kg/m^2^). Blood pressure was measured with a mercury sphingomanometer after 5 minutes in the sitting position.

The presence of macrovascular comorbidities was defined as past myocardial infarction or cardiac intervention (percutaneous intervention or bypass surgery), history of cerebral ischemia (transient ischemic attack, stroke) or peripheral arterial disease (ABI <0.90 or percutaneous intervention or peripheral bypass surgery).

### Biochemical analyses

A blood sample was taken at both visits after 12-14 hours of overnight fast in sodium-EDTA tubes and stored at -80 °C.

Patients were asked to omit antidiabetic treatment on the evening before their visit. Blood glucose before and after a standard oral glucose (75g) tolerance test was directly measured from whole-blood samples from a finger stick (fasting, 1-h and 2-h samples) by using plasma referenced reflection photometry (Reflotron Sprint; Roche, Basel, Switzerland). Insulin resistance was estimated from fasting glucose and C-peptide concentrations by using a computer-based homeostasis model assessment system (HOMA2-IR) provided by the Oxford Centre for Diabetes, Endocrinology, and Metabolism (http://www.dtu.ox.ac.uk/homa).

HbA1c was measured with the DCA 2000 (Bayer Diagnostics, Elkhart, USA) according to the manufacturer’s instructions. Measurement of urinary albumin and creatinine was done in spot urine with the DCA 2000. Microalbuminuria was defined as presence of urinary albumin/creatinine ratio >2.5 mg/mmol, macroalbuminuria was defined as presence of an urinary albumin/creatinine ratio >25 mg/mmol [19]. Cholesterol was measured by an enzymatic colorimetric test using cholesterol esterase and cholesterol oxidase, triglycerides were determined by a colorimetric reaction with iodonitrotetrazolium chloride after enzymatic hydrolysis (modular P lab analyzer, Roche, Switzerland). HDL measurement was done by a homogeneous enzymatic test (Cobas Integra lab analyzer, Roche, Switzerland). LDL was calculated with the Friedewald formula [20].

Nondenaturing polyacrylamide GGE (gradient gel electrophoresis) of plasma was performed at 10-14 °C in 2-16% polyacrylamide gradient gels. Gels were subjected to electrophoresis for 24 h at 125 V in tris borate buffer (pH 8.3) as described elsewhere [21]. Gels were fixed and stained for lipids in a solution containing Oil Red O in 60% ethanol at 55 °C. Gels were placed on a light source and photographed using a Luminescent Image Analyzer, LAS-3000 of Fujifilm. Migration distance for each absorbance peak was determined and the molecular diameter corresponding to each peak was calculated from a calibration curve generated from the migration distance of size standards of known diameter, which includes carboxylated latex beads (Duke Scientific, Palo Alto, CA), thyroglobulin and apoferritin (HMW Std, Pharmacia, Piscataway, NJ) having molecular diameter of 380 nm, 170 nm and 122 nm, respectively, and lipoprotein calibrators of previously determined particle size. LDL subclass distribution (LDL I, IIA, IIB, IIIA, IIIB, IVA and IVB) as percentage of total LDL was calculated as previously described [21].

From frozen serum, C-peptide was measured by using radioimmunoassay (RIA) (IRMA-C-PEP; CIS Bio International, Bagnols-sur-Cèze Cedex, France), adiponectin and resistin were measured by using ELISA (DRP300 and DRSN00 ELISA Kits; R&D Systems, Minneapolis, MN).

### Vascular assessment

IMT (Intima media thickness) was measured in both common carotid arteries using high-resolution B-mode ultrasound with a 9-3 MHz linear array transducer (iU22, Philips, Best, Netherlands) as previously described [22,23]. Reference point for the measurements of IMT was 1 to 2 cm proximal to the dilatation of the carotid bulb. The far wall was scanned from an anterolateral direction and IMT was automatically computed by the ultrasound software (QLAB, Philips, Best, Netherlands). Mean IMT was calculated as the mean of both common carotid arteries.

Forearm blood flow of the brachial artery is increased in response to transient hyperaemia and was studied using high-resolution ultrasound of the brachial artery [24,25]. Patients were lying in supine position for 10 minutes at rest before the measurement was started. The right arm was fixed in extended, relaxed position to allow correct analysis of the brachial artery 2-5 cm above the antecubital fossa. The brachial artery was visualized longitudinally using a 17-5 MHz linear array ultrasound transducer (iU22, Philips, Best, Netherlands); B-mode and pulsed Doppler spectral curve were recorded. A cuff placed around the forearm distal to the imaged artery segment was inflated to about 30 mmHg of above the systemic systolic arterial pressure for five minutes. Maximal brachial artery diameter was determined and the mean value of vessel calibre was calculated from six single measurements made before the cuff inflation and from six records taken every minute after cuff release. The peak value diameter acquired during ischemia-induced hyperaemia was used for the evaluation of the percentage FMD (flow mediated vasodilatation) (maximum diameter - baseline diameter) / baseline diameter x 100%.

### Statistical analyses

Data are expressed as arithmetic means ± SDs for normally distributed variables and as geometric means ± SDs for non-normally distributed data. For the analysis of independent categorical frequency data, the χ^2^ test was applied, and for related categorical frequency data, a McNemar test was performed. For comparison of continuous variables in two independent groups, the Mann–Whitney test was used, for related samples, the Wilcoxon test was applied (Multiple). linear regression was used for the testing of correlations. A value of p < 0.05 was considered significant. Outliers were only excluded if identified by both the Dixon’s and the Grubbs’ test with high significance (p<0.01). The statistical analyses were performed using SPSS 20 software (IBM, New York, USA) for Windows (Microsoft, Redmond, USA).

## Results

### Demographic characteristics

Fifty-nine white males were included in the study and assessed for baseline characteristics, 39 (66%) of them were seen again for a second assessment; the 20 remaining subjects were excluded because they refused further participation after the first assessment (5 subjects) or they had to be excluded due to the study’s exclusion criteria (low BMI 4, high triglycerides 4, high creatinine 4, insulin therapy 2, malignant disease 1). The mean time between the first and the second assessment was 24.9 ± 1.2 months. Baseline anthropometric and biochemical data of all patients are summarized in Table 1. 34% of all participants were known to have peripheral arterial disease, 19% had a history of myocardial infarction and 4% had a history of stroke. Diabetic retinopathy was known in 5% of participants and diabetic neuropathy in 18%. Nephropathy as assessed by microalbuminuria (albumin/creatinine ratio) at baseline was present in 32%. 73% of study participants received oral antidiabetic drugs, with metformin being the mostly prescribed substance (in 77% of treated participants). 59% of the patients were treated with a statin.

**Table 1:** Baseline characteristics of study participants.

Characteristic	Baseline
Age (y)	63.0 ± 12.2
Duration of diabetes or prediabetes (y)	7.3 ± 7.0
BMI (kg/m^2^)	30.5 ± 6.5
Waist-to-Hip Ratio	1.01 ± 0.05
Systolic blood Pressure (mmHg)	137.3 ± 16.6
Diastolic blood pressure (mmHg)	78.6 ± 8.5
Total Cholesterol (mmol/l)	4.4 ± 1.0
HDL Cholesterol (mmol/l)	1.2 ± 0.4
Triglycerides (mmol/l)	1.8 ± 1.0
LDL Cholesterol (mmol/l)	2.4 ± 0.9

Mean ± standard deviation

### Assessment of LDL particle size

Analysis of LDL particle size by GGE revealed a mean particle size of 262.4 ± 8.5 nm, with 25.4% of the subjects exhibiting a pattern B phenotype. The proportion of small dense LDL particles (class III/IV) was 39.0 ± 11.2% at baseline and 43.6 ± 11.5% at follow up. In 68% of patients, this proportion had increased during follow-up. Patients with known previous myocardial infarction had a significantly higher proportion of sdLDL particles at baseline (49.8% vs. 37.5% in patients without history of myocardial infarction, p=0.007). With regard to mean LDL particle size and distibution there was no difference at baseline and at follow-up when study participants treated with statin therapy were compared with those who had no lipid lowering therapy. In contrast, LDL concentration was significantly lower in treated patients at baseline (2.1 ± 0.9 mmol/l vs. untreated 2.9 ± 0.7 mmol/l, p<0.001) and after follow-up (2.1 ± 1.0 mmol/l vs. untreated 2.9 ± 1.0 mmol/l, p=0.005). There was no association of changes in BMI with changes in sdLDL particles during follow-up.

### LDL particle size distribution and measures of arterial disease

**Figure 1:**
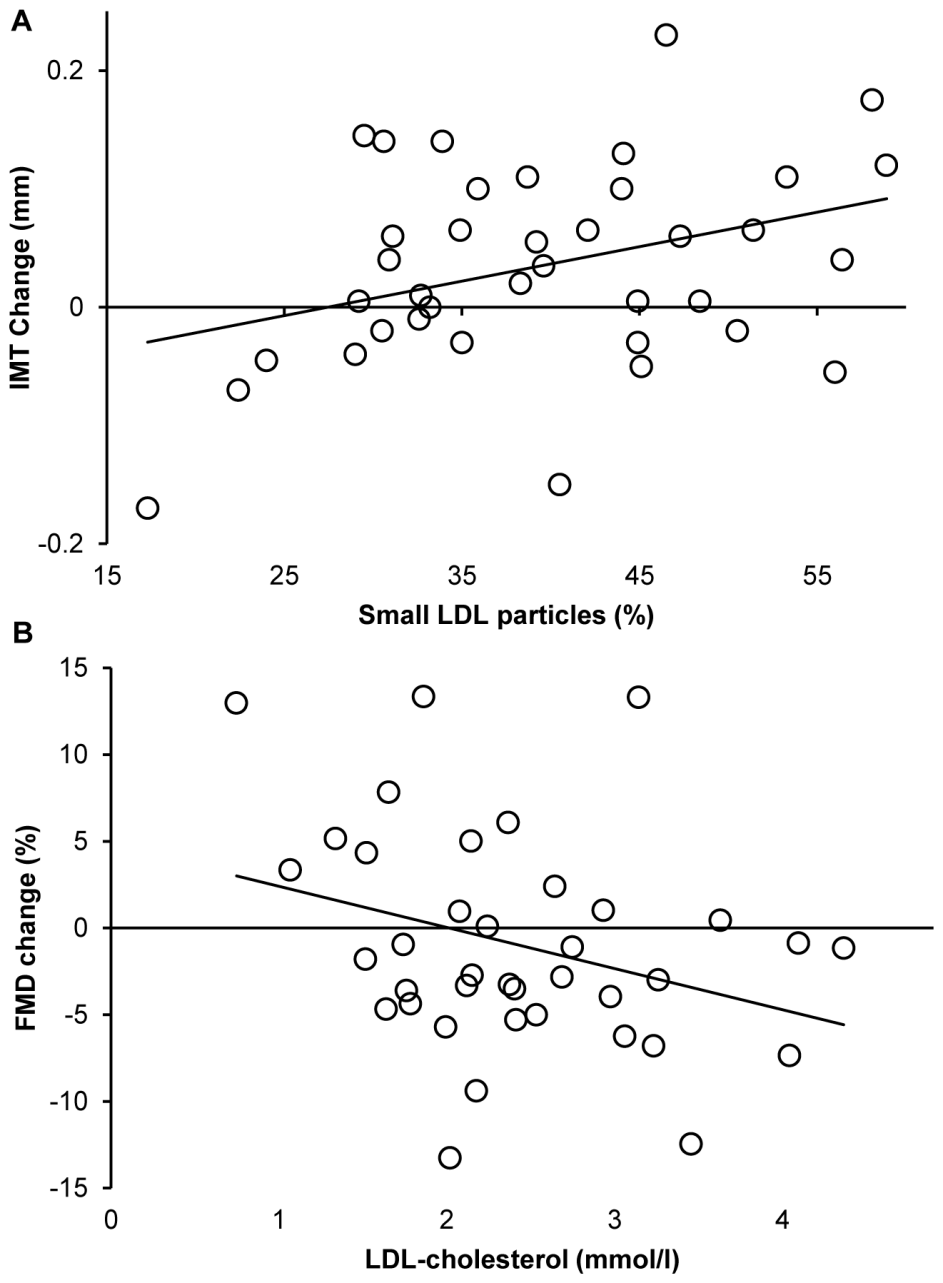
Prediction of changes in IMT and FMD bei sdLDL and LDL-C. Changes in IMT during the 2 year follow-up are shown in relation to the proportion of small, dense LDL particles at baseline (a). Changes in FMD during follow-up are shown in relation to the proportion of LDL-C levels at baseline (1B), p<0.05.

Intima-media thickness (IMT) and flow mediated dilation (FMD) were assessed as surrogate markers to detect morphological and functional changes of the arterial system. IMT was 0.68 ± 0.14mm at baseline and was significantly larger at the second assessment (0.73 ± 0.10mm, p=0.01). FMD, which was 7.2 ± 5.3% at baseline, was 5.7 ± 4.5% after 2 years (ns). The change in IMT correlated with the proportion of sdLDL particles at baseline (R^2^=0.124, p=0.03, Figure 1a). Analysis using multiple linear regression revealed that small dense LDL particles remained a predictor of IMT progression even when including HbA1c, BMI, age and systolic blood pressure into the model (p=0.003, table 2). In this analysis, HbA1c, BMI and systolic blood pressure did not correlate with IMT progression, however a higher age (which correlated with higher IMT at baseline, p=0.004) predicted less IMT progression (table 2). Furthermore, none of the traditional lipid parameters (Total and LDL-cholesterol, HDL-cholesterol, triglycerides) at baseline was associated with changes in IMT. Deterioration of FMD during follow-up was predicted by the level of LDL-cholesterol at baseline (R^2^=0.103, p=0.049, Figure 1b) but not the proportion of sdLDL particles, other conventional lipid parameters or age, HbA1c or BMI. High systolic or diastolic blood pressure was not a predictor for a reduced FMD, however, increasing systolic blood pressure during the 2 years of follow-up was associated with worsening of FMD (R^2^=0.102, p=0.05).

**Table 2:** Multiple linear regression was performed to assess the correlation of changes in IMT with baseline measurements of sdLDL particles, age, BMI, systolic blood pressure and HbA1c.

		95% Confidence Interval		
Parameter	Coefficient	Lower	Upper	p-Value
sdLDL particles	0.004	0.001	0.006	0.003
Age	-0.002	-0.004	0.000	0.04
BMI	-0.004	-0.008	0.000	0.08
Systolic blood pressure	0.000	-0.002	0.001	0.74
HbA1c	0.016	-0.028	0.061	0.47

### LDL particle size distribution and insulin resistance

**Figure 2:**
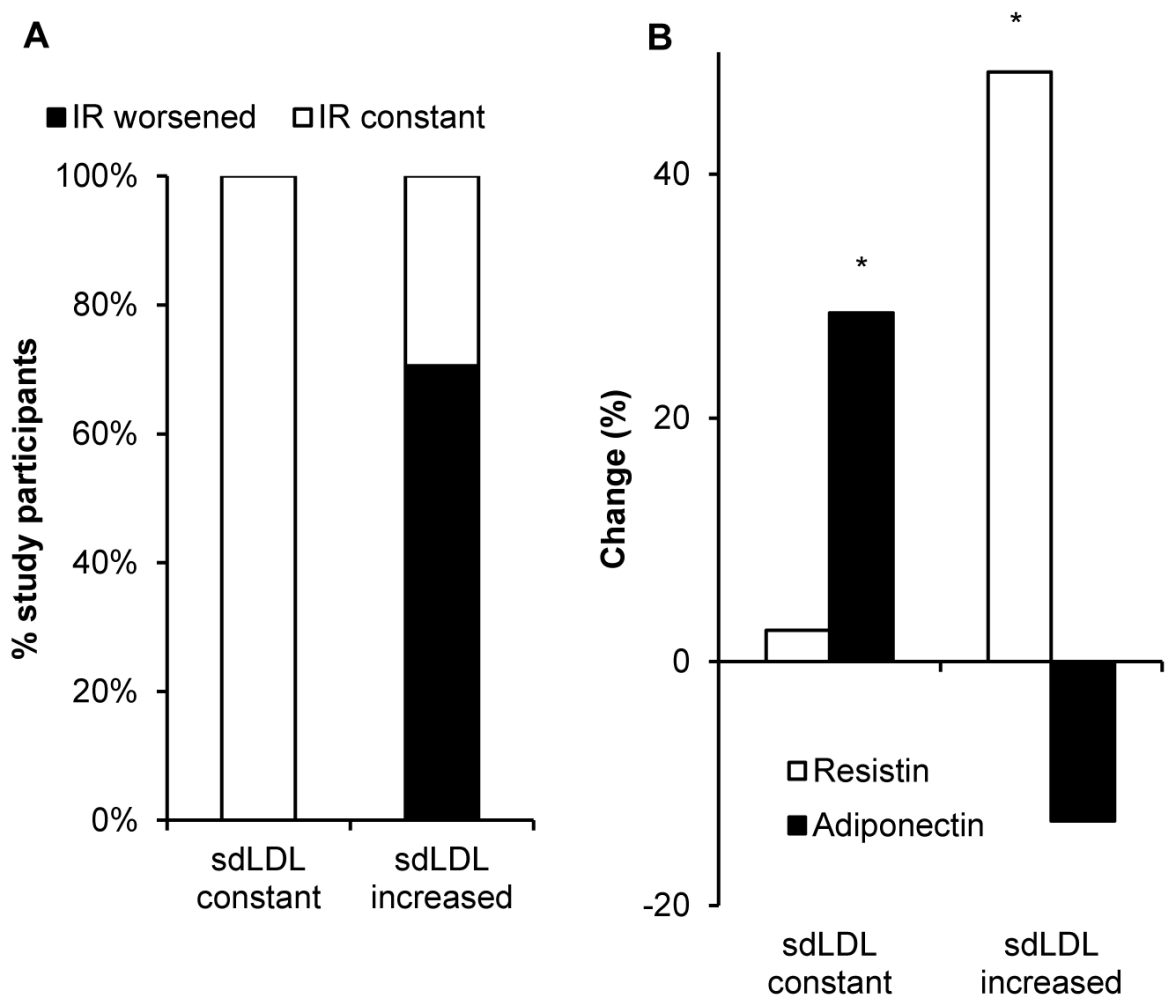
Changes in HOMA2 and resistin / adiponectin levels depend on changes in the proportion of sdLDL particles. The Proportion of patients (%) with constant or improved insulin resistance (white) or worsened insulin resistance (black) (assessed by HOMA2 during 2 years of follow-up) is shown for those patients with and those without an increase in small, dense LDL particles (2A, p<0.05). Changes (%) of resistin and adiponectin levels during the 2 years of follow-up are shown in in patients with and those without an increase in small, dense LDL particles (2B). * Significantly different from baseline, p<0.05.

Insulin resistance and glucose control were assessed by measuring fasting glucose and HbA1c levels as well as on the basis of a glucose tolerance test and HOMA2 calculations. Measurements at the first and second visit are given in Table 3. HbA1c significantly increased between the first and the second assessment by 0.3 ± 0.7% (p=0.03). At baseline, C-Peptide concentration and insulin resistance both correlated with the proportion of sdLDL particles (p=0.02 and p=0.04, respectively). The association of HOMA2 estimated insulin resistance with sdLDL particles was still present at the second visit (p=0.02). Importantly, there was neither an association of HOMA2 with HbA1c at baseline nor at follow-up. There was no worsened insulin resistance in any of the patients in whom the proportion of sdLDL particles did not rise during follow-up, however, worsened insulin resistance occurred in 70.6% of participants displaying an increased proportion of small, dense LDL particles at follow-up (Figure 2a, p = 0.04). In contrast, there was no relationship between worsening of glycemic control (increase in HbA1c) and increased small LDL particles (HbA1c +0.2 ± 0.8 vs. +0.4 ± 0.6 in patients without increased sdLDL proportion, ns). Accordingly, there was no association between changes in HbA1c and insulin resistance (HbA1c +0.2 ± 0.5 vs. -0.2 ± 0.5 in patients with and without increase in HOMA2, respectively, ns).

**Table 3:** Measures of glucose control, insulin resistance and adipokines in patients attending both visits.

Characteristic	Baseline	Follow-up
Fasting glucose (mmol/l)	6.6 ± 1.3	6.9 ± 1.6
Glucose 60min after 75g oral glucose (mmol/l)	13.4 ± 3.0	12.3 ± 3.5
Glucose 120min after 75g oral glucose (mmol/l)	12.5 ± 4.2	10.6 ± 3.8
HbA1c (%)	6.4 ± 0.6	6.6 ± 0.9*
C-Peptide (nmol/l)	1.46 ± 0.77	1.69 ± 0.81
HOMA2-IR	3.36 ± 1.68	4.01 ± 1.88
Adiponectin (μg/ml)	4.98 ± 3.67	5.25 ± 3.58
Resistin (ng/ml)	15.4 ± 7.5	23.1 ± 16.4*

Mean ± standard deviation. * Significantly different from baseline, p<0.05

Regarding conventional lipid parameters, HDL-cholesterol also correlated with HOMA2 estimated insulin resistance at baseline and after 2 years (p=0.005 and p=0.004, respectively) whereas triglyceride levels and the triglyceride/HDL-C ratio only correlated with HOMA2 at baseline (p=0.02 and p=0.001, respectively), but not after follow-up. Changes in HOMA2 during follow-up were not different between patients with increased or stable HDL-cholesterol, triglyceride or triglyceride/HDL-C ratio.

### LDL particle size distribution and levels of adiponectin / resistin

Serum levels of adiponectin and resistin were assessed at both visits (table 3). In the whole cohort, resistin levels increased (p=0.001) and were closely correlated with the number of sdLDL particles at the first (p=0.02) and second (p<0.001) visit. In patients showing an increase in the proportion of sdLDL particles, resistin concentrations also increased (p<0.001), which was not the case in those patients whose sdLDL particle number did not rise during follow-up (Figure 2b); the increase in resistin directly correlated with the increase in sdLDL particle number (R^2^=0.149, p=0.01). On the other hand, serum adiponectin increased only in patients without increasing sdLDL particles (p=0.006), but not in those with more sdLDL particles at follow-up. To assess relevant effects of possible subclinical inflammation on resistin levels, measures of C-reactive protein (CRP) were included in the analysis in a subset of 16 patients at the first and / or second visit. Resistin levels still correlated with sdLDL particles (p=0.03), but not with logCRP levels (p=0.55) when this parameter was included using multiple linear regression.

No deaths and only one major cardiovascular event (stroke) occurred during follow-up. 

## Discussion

This prospective cohort study demonstrates the value of LDL particle size measurements in the prediction of changes in metabolic status and cardiovascular disease in patients with diabetes or prediabetes.

The fraction of small LDL particles at baseline predicted changes in intima-media thickness that occurred during the following two years, with a larger fraction of sdLDL particles being associated with a larger increase in IMT. Whereas a correlation of IMT with actual LDL particle size was demonstrated by many earlier studies including our own data [7,26], the predictive value of LDL size with regard to IMT in dysglycemic patients has not been investigated in prospective long-term studies. Of interest, other parameters as HbA1c, BMI, or systolic blood pressure were not associated with the extent of IMT increase in the present study. This might be partly due to the inclusion criteria of the study, which narrowed the spread of these parameters within the cohort and thereby limited the possibility to determine their predictive value. Further, the superiority of LDL particle size in comparison to other conventional risk factors may also be due to its comprehensive character – as the number of small LDL particles is influenced by age, weight, body composition and also metabolic control and may therefore provide more precise information. Higher age at baseline predicted less IMT progression during follow-up, this is most probably because of an association of higher age with an already significantly higher IMT at baseline.

Of interest, changes in flow mediated dilation were only predicted by the LDL-C concentration, but neither by sdLDL levels or other lipid parameters nor by age, BMI or HbA1c, whereas changes in IMT were only predicted by the proportion of sdLDL particles, but not by LDL-C levels. It is, thus, tempting to assume that changes in the arterial structure (as IMT) are best predicted by a factor that robustly reflects the organism’s cardiovascular risk status, whereas factors that are easily modifiable (mostly by therapeutic interventions) are more likely to predict functional characteristics (as FMD).

HbA1c levels provide insufficient information on cardiovascular risk in patients with diabetes or prediabetes, and this is reflected by the complete lack of an association of this parameter with insulin resistance or changes in insulin resistance in this study. HbA1c adequately reflects glycemia and metabolic control achieved by antihyperglycemic therapy and may predict a large proportion of the cardiovascular risk in patients with type 1 diabetes, where hyperglycemia is the predominant mediator of cardiovascular damage. However, additional effects mediated by insulin resistance (e.g. altered lipid metabolism) are not reflected by HbA1c. In contrast, in addition to the direct association of absolute HOMA2 values with sdLDL particles at the first and second visit (confirming our previous cross-sectional data in another study population [15]), changes in insulin resistance also differed clearly between patients with and without an increase in sdLDL particles between the two visits. While triglyceride and HDL-C concentration as well as their ratio were also associated with insulin resistance as previously established [27,28], changes of HOMA2 were independent of changes in HDL-C or triglyceride levels. Thus, alterations in the proportion of sdLDL particles seem to be a very early and accurate predictor of alterations in insulin resistance.

These observations are supported by concordant data on serum adiponectin and resistin concentrations. Decreased levels of adiponectin are known to be a reliable predictor of insulin resistance and progression to type 2 diabetes [29,30]. In this study, patients with stable or decreasing sdLDL number showed a rise in adiponectin concentration during follow-up, whereas adiponectin levels did not change if sdLDL particles increased. Furthermore, a tight correlation of resistin levels with the proportion of sdLDL particles could be observed at both visits. Resistin concentration increased only in subjects who also displayed an increase in sdLDL particles, with a direct correlation of the increase in sdLDL particles and resistin levels. Although the discussion about metabolic effects of resistin in humans remains controversial, its association with parameters of the metabolic syndrome has been repeatedly documented [31,32]. Taken together, the concomitant changes in adiponectin and resistin concentration strengthen the argument for a predictive value of changes in the proportion of small dense LDL particles concerning the metabolic status of adipose tissue, which is one of the most important determinants of the further course of insulin resistance and the associated cardiovascular risk.

The strength of this study is its prospective longitudinal character that allowed assessing the predictive value of small dense LDL particles in a well defined cohort of male patients with diabetes and prediabetes. Further, due to the single centre design of the study, it was possible that all clinical and biochemical assessments were performed at the same place and by the same investigators, therefore limiting possible inter-observer biases. This was of particular importance with respect to the measurements of intima media thickness and flow-mediated dilation. A limitation is the relatively small sample size. Further, standardization of therapeutic interventions regarding hyperglycemia and hypercholesterolemia were not part of the study protocol, and therefore established glucose- and cholesterol-lowering therapies may have influenced study measures. However, glucose lowering treatment was suspended the night before the visits. Furthermore, sdLDL particle size distribution was assessed separately in patients with and without statin therapy, without detectable differences.

In conclusion, this study provides evidence that the proportion of small, dense LDL particles and changes in this proportion predict changes in intima media thickness and insulin resistance, and are closely associated with other determinants of an adverse metabolic status. Therefore, this parameter offers the possibility to extend the individual risk assessment beyond the limitations of traditional risk markers in patients with dysglycemia. 
